# Supplementary figures and images for: Microhomology-mediated circular DNA formation from oligonucleosomal fragments during spermatogenesis
Source: eLife. 2023 Oct 17;12:RP87115. doi: 10.7554/eLife.87115 (PMC10581685; doi:10.7554/eLife.87115)

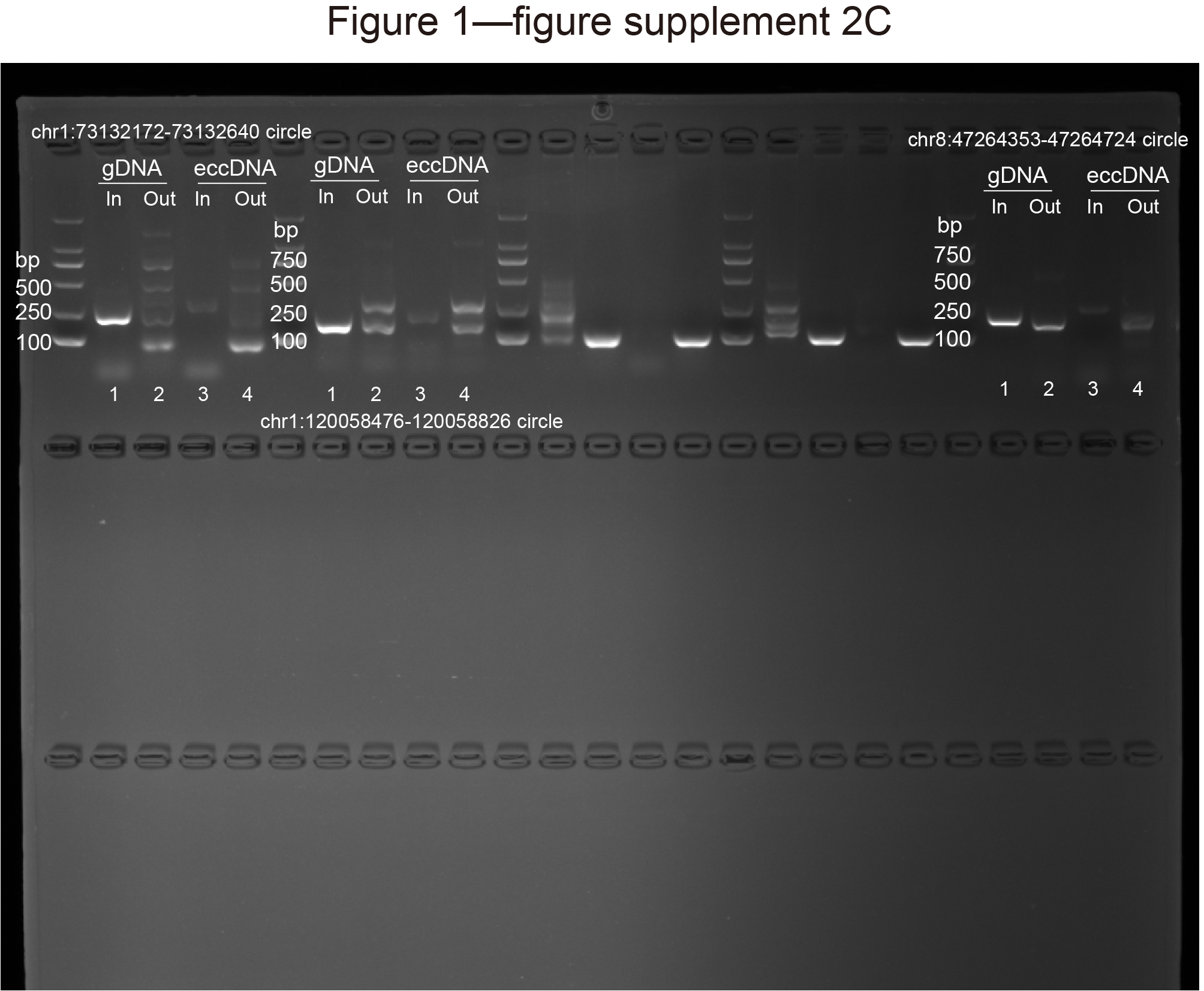

Supplement: Figure 1—figure supplement 2—source data 1. [file elife-87115-fig1-figsupp2-data1.zip › Figure 1-figure supplement 2C-source data with label.jpg]
